# Traditional Chinese medicine suppressed cancer progression by targeting endoplasmic reticulum stress responses: A review

**DOI:** 10.1097/MD.0000000000032394

**Published:** 2022-12-23

**Authors:** Fan Xia, Suling Sun, Li Xia, Xiuli Xu, Ge Hu, Hongzhi Wang, Xueran Chen

**Affiliations:** a Hefei Cancer Hospital, Chinese Academy of Sciences, Hefei, Anhui, China; b Anhui Province Key Laboratory of Medical Physics and Technology, Institute of Health and Medical Technology, Hefei Institutes of Physical Science, Chinese Academy of Sciences, Hefei, Anhui, China.

**Keywords:** apoptosis, endoplasmic reticulum stress (ERS), invasion and metastasis, traditional Chinese medicine (TCM), tumor treatment

## Abstract

Cancer has a high morbidity and mortality; therefore, it poses a major global health concern. Imbalance in endoplasmic reticulum homeostasis can induce endoplasmic reticulum stress (ERS). ERS has been shown to play both tumor-promoting and tumor-suppressive roles in various cancer types by activating a series of adaptive responses to promote tumor cell survival and inducing ERS-related apoptotic pathways to promote tumor cell death, inhibit tumor growth and suppress tumor invasion. Because multiple roles of ERS in tumors continue to be reported, many studies have attempted to target ERS in cancer therapy. The therapeutic effects of traditional Chinese medicine (TCM) treatments on tumors have been widely recognized. TCM treatments can enhance the sensitivity of tumor radiotherapy, delay tumor recurrence and improve patients’ quality of life. However, there are relatively few reports exploring the antitumor effects of TCM from the perspective of ERS. This review addresses the progress of TCM intervention in tumors via ERS with a view to providing a new direction for tumor treatment.

## 1. Introduction

Cancer is a major disease that threatens the lives and health of people worldwide. With 19.29 million new cancer cases and nearly 10 million cancer-related deaths worldwide in 2020, the global incidence and mortality of cancer are vital health concerns.^[1,2]^ Western medicine is the main cancer treatment, but the overall results are not satisfactory.

The endoplasmic reticulum is an important site for protein modification, folding, assembly, and secretion in eukaryotic cells, as well as for intracellular calcium ion storage. Unfolded and misfolded proteins accumulate in the lumen of the endoplasmic reticulum, causing endoplasmic reticulum stress (ERS). ERS is relieved by the unfolded protein response (UPR). The glucose regulated protein 78 (GRP78), also known as lumenal-binding protein (BiP), as an endoplasmic reticulum homeostatic receptor, dissociates from the 3 UPR effectors protein kinase R (PKR)-like endoplasmic reticulum kinase (PERK), IRE1 and activating transcription factor (ATF)-6, and instead binds to the new unfolded proteins, while the activated free 3 transmembrane receptor proteins stimulate the UPR signaling cascade. Unlike normal cells, tumor cells are uniquely dependent on ERS for survival due to the local microenvironment and high demands on protein synthesis. However, when ERS persists and is too strong, it can induce apoptosis in tumor cells. Therefore, targeted inhibition or enhancement of ERS in tumor cells abrogates the malignant biological behavior of tumor cells and exerts an anti-tumor effect.^[3,4]^

Some clinical antitumor broad-spectrum chemotherapeutic drugs originate from natural sources, such as paclitaxel (PTX), which is extracted from the Pacific yew. Chinese medicines originate from nature and have been used for thousands of years. These medicines have a certain efficacy and advantages such as mild side effects and being inexpensive and easy to use. Chinese herbal medicines are often used in clinical practice as compound tonics; however, the mechanisms by which they exert antitumor effects are unclear. This article focuses on the progress of traditional Chinese medicine (TCM) intervention in tumors via ERS to provide research ideas and a theoretical basis for antitumor treatment.

## 2. Tumor cell proliferation

There are 2 key apoptosis pathways: exogenous signaling pathways, also known as death receptor pathways, which are triggered by cytokines, hormones, pathogenic microorganisms, and drugs, including FAS/FASL, TRAILR/TRAIL, and tumor necrosis factor (TNF)-R1/TNF pathways; and endogenous signaling pathways, which are apoptotic signals generated within the cell, such as reactive oxygen species (ROS), cytochrome C, and calcium with ERS. Most apoptotic signals converge on the mitochondria, which then trigger downstream apoptosis-related regulation that may or may not be dependent on the caspase family.^[5]^ ERS is a protective response of cells against stress, and moderate ERS can contribute to cell survival and promote tumor progression. However, excessive, sustained, and intense ERS induces apoptosis. Several studies have found that ERS can promote apoptosis in tumor cells by disrupting intracellular Ca^2 + ^to activate caspases through IRE1α to induce apoptosis signal-regulating kinase 1 (ASK1), and tumor cell apoptosis via c-Jun NH2-terminal kinase (JNK) and p38 signaling pathways.^[6]^ In addition, PERK, ATF6 and IRE1a induce deoxyribonucleic acid damage inducible transcript 3 homologous protein (DDIT3, also known as CHOP) transcription, which in turn induces apoptotic gene (e.g., DR5, TRB3, PUMA and BIM) expression and caspase activation, such as caspase-12 or caspase-4.^[7]^ Furthermore, ERS dissociates TNF receptor-associated factor (TRAF)-2 from caspase-12 and activates apoptotic effector enzymes such as caspase-12, caspase-9 and caspase-3, which ultimately induce apoptosis.^[8]^

### 2.1. Head and neck cancer

ChaC glutathione specific gamma-glutamylcyclotransferase 1 (CHAC1) is also the downstream target of the ATF4-CHOP axis. Wang et al found that glaucocalyxin A activates ROS-mediated ATF4/CHOP/CHAC1 signaling, thereby inhibiting proliferation and inducing apoptosis in the oral squamous cell carcinoma cell lines SCC25 and CAL27.^[9]^ Tetrandrine can induce cell apoptosis in NPC-TW 039 human nasopharyngeal carcinoma cells by activating ERS stress related protein GADD153 and the expression of GRP78 through elevated intracellular Ca^2+^.^[10]^

### 2.2. Digestive tract tumor

#### 2.2..1. Esophageal cancer.

Tan-IIA can induce Eca-109 cell apoptosis by decreasing BiP expression and then activating CHOP, and the ERS pathways may play an important role in Tan-IIA-induced Eca-109 cell apoptosis.^[11]^

#### 2.2..2. Gastric cancer.

Gu Q et al found that Wogonoside can activate the ERS sensors IRE1a. The activated IRE1a interacts with the scaffold molecule TRAF2 and subsequently recruits ASK1. Then, the IRE1a-TRAF2-ASK1 complex activates JNK, which participates in cell apoptosis by regulating the phosphorylation and activation of the BCL2 apoptosis regulator (Bcl-2) family proteins. Thus, the inositol-requiring enzyme-1α (IRE1α)-TRAF2-ASK1 signaling pathway plays a key role in ERS and baicalein-induced apoptosis.^[12]^

#### 2.2..3. Liver cancer.

*Cyperus amuricus* can induce cell apoptosis in Hep3B human hepatoma cells, by activating the GRP78/BiP-phosphorylated eIF2a-CHOP pathway through elevated intracellular Ca^2+^.^[13]^ Muscone treatment markedly increased the phosphorylation levels of PERK and eukaryotic translation initiation factor 2 subunit alpha (eIF2α) proteins, and the expression levels of ATF4 and deoxyribonucleic acid damage inducible transcript 3 (DDIT3). DDIT3 subsequently accumulates in the nucleus, promoting apoptosis and inhibiting the expression of the anti-apoptotic protein, Bcl-2. Muscarinic acid induces apoptosis in human hepatocellular carcinoma cells (HCC) HepG2, through the PERK/ATF4/DDIT3 pathway and also inhibits the growth of transplanted HCC subcutaneous tumors in vivo.^[14]^ One study found that celastrol can target ERS to promote apoptosis in HepG2, Bel7402 cells via the IRE1α-X-box binding proteins 1 (XBP1s) pathway and PERK-elF2α-ATF4-CHOP pathway through upregulation of GRP78/BiP expression.^[15]^ Salidroside, the effective component of rhodiola can promote apoptosis of human HepG2 cell lines by enhancing PERK and eIF2a, upregulation of CHOP and ATF6.^[16]^ 5,2’,4’-trihydroxy-6,7,5’-trimethoxyflavone (TTF1) is the primary anticancer constituent of the TCM plant *Sorbaria sorbifolia*. Xiao B et al found that small-molecule TTF1 nanoparticles (TTF1-NPs) increased the expression of other ERS-related signaling molecules (PERK, IREα, and ATF6) and apoptosis mediators (JNK, p-JNK, and CHOP) in TTF1-NP-treated HepG2 cells, which show that TTF1-NP-induced apoptosis is at least in part mediated by the ERS pathway.^[17]^ However, it is important to note that some other studies found some TCM treatment in liver cancer that will kill the tumor by inhibiting ERS. For example, caudatin, a steroid from *Radix bupleurum*, can exert antitumor effects in the diethylnitrosamine-induced cirrhotic rat model with HCC by inhibiting the PERK-ATF4-eIF2α and ATF6 pathways to suppress UPR.^[18]^ Wang J et al found that catechins direct targeting to inhibit poly-(adenosine diphosphate-ribose) polymerase 16 activity and suppress PERK and eIF2α phosphorylation, by inhibiting protective ERS-induced apoptosis in cancer cells.^[19]^ These results suggest that the antitumor effects of herbal medicines are complex, which may be related to different cancer types and drug components.

#### 2.2..4. Colorectal cancer.

Shikonin inhibits the proliferation of HCT-116 and HCT-15 colorectal cancer cells in vitro and in vivo by increasing BiP, activating the PERK/elF2α/ATF4/CHOP and IRE1α/JNK signaling pathways, upregulating the anti-apoptotic protein Bcl-2, and increasing the expression of caspase-3/9.^[20]^ One study showed that gambogenic acid induces apoptosis and inhibits cell proliferation in HCT116 colon cancer cells by promoting the phosphorylation of eIF2α, IRE1α-XBP1s and PERK pathways through Aurora A.^[21]^ In addition, another study found that a purified resin glycoside fraction from pharbitidis semen promote apoptosis in HT-29 and HCT-116 cells via ER stress.^[22]^

### 2.3. Gynecologic urogenital tumors

ER stress can participate in the apoptosis induced by Saikosaponin-A in HeLa cells as indicated by the upregulation of GPR78, CHOP and caspase-12 expression.^[23]^ Dihydrotanshinone I acts as a disulfide-isomerase like protein ERp57 inhibitor, leading to accumulation of unfolded proteins, activation of the PERK pathway, and subsequent apoptosis in MDA-MB-231 breast cancer cells.^[24]^

### 2.4. Non-small cell lung cancer (NSCLC)

Chen et al found kushenol Z could promote apoptosis in A549 and NCI-H226 NSCLC cell line by upregulation CHOP and activate caspase-7 and caspase-12 to trigger ERS.^[25]^ Evodiamine increases TRAF2, ASK1, and p-JNK levels and promotes apoptosis in A549 and NSCLC cells via the IRE1α-TRAF2-ASK1 signaling pathway.^[26]^ Yang B et al found target ERS to inhibit tumor growth in Lewis lung cancer transplantation tumors in C57BL/6 tumor-bearing mice via the IRE1α-XBP1 pathway.^[27]^

### 2.5. Others

Liu Y et al found that comfrey induces apoptosis and protective autophagy by activating ROS and mediating ERS and p38 signaling in A375 human melanoma cells.^[28]^ Li S et al found that psoralen induces apoptosis in human osteosarcoma cells by upregulating ATF6 and CHOP and downregulating Bcl-2.^[29]^ Artemisinin acts on the PERK-ATF4-HSPA5 pathway and the ATF4-CHOP-CHAC1 pathway in ERS to induce ferroptosis in Burkitt lymphoma.^[30]^ Martinotti et al found that catechin induces ATF4, XBP1 shear, CHOP, and EDEM expression by increasing GRP78 expression, converting adaptive UPR into an ERS response that promotes apoptosis in recalcitrant malignant mesothelioma cell lines.^[31]^ In addition, another study found that asperuloside induces apoptosis in human leukemia cell lines including U937 and HL-60 cells by phosphorylating ERS receptors (IRE1α, PERK, ATF6) via targeting GRP78.^[32]^

## 3. Tumor cell autophagy

Cellular autophagy is the process by which eukaryotic cells use lysosomes to degrade cytoplasmic proteins and damaged organelles under the regulation of autophagy-related genes. Cellular autophagy plays a dual role in preventing damage and promoting cell survival in the absence of oxygen and nutrient deficiency and in blocking cell survival and growth.^[33]^ Therefore, cellular autophagy can mediate drug resistance and promote tumor progression, but it can also induce cell death. Cellular autophagy is closely linked to ERS. UPR activation may activate autophagy to remove misfolded proteins accumulated in the ER lumen, inducing conversion of LC3 I to LC3 II and the formation of autophagosomes, leading to protective autophagy and alleviating ERS.^[33,34]^ ERS also activates autophagy by negatively regulating the Akt serine/threonine kinase (Akt)/TSC/mechanistic target of rapamycin kinase pathway and positively regulating the IRE1α-JNK pathway.^[35]^ However, excessive ERS induces autophagy in tumor cells, which eventually leads to cell death. The fate of cells under ERS may stem from shifts in the balance between UPR and autophagy.

### 3.1. NSCLC

Ginsenoside can activate ERS through the ATF4-CHOP-AKT1-mechanistic target of rapamycin kinase pathway to upregulate autophagic flux and induce autophagic death in NSCLC cells.^[36]^ Guangsangon E is a Diels–Alder adduct isolated from mulberry leaves that activates UPR to induce cell autophagy and death in A549 human NSCLC cells.^[37]^

### 3.2. Colorectal cancer

In HT-29 colon cancer cells, the ERS signaling proteins GRP78, p-PERK/PERK, p-elF2α/elF2α, and CHOP were upregulated and apoptosis was increased after serpentine treatment.^[38]^ Inhibition of cellular autophagy using 3-methyladenine suppresses apoptosis induced by serpentine via the ERS pathway. Mitigation of ERS with a 4-phenylbutyric acid inhibitor also attenuates serpentine-induced apoptosis and autophagy. Cryptotanshinone inhibits the growth of HCT116 colorectal cancer cells through ERS-mediated autophagy.^[39]^

### 3.3. Gynecologic urogenital tumors

Li H et al found that cucurbitacin-I induced both strong ERS response and strong autophagy levels in SKOV3 human ovarian cancer cells. Cucurbitacin-I activated 2 of the 3 ERS pathways, IRE1α and PERK, as well as CHOP, but not ATF6α pathway. The excessive ERS also triggered autophagy and autophagic death. There are significantly reduced levels of autophagy in cells treated with ERS inhibitors.^[40]^

Thus, these results suggest that ERS is closely linked to cellular autophagy, and some TCM treatment can trigger autophagy and autophagic death.

## 4. Tumor cell invasion and metastasis

ERS is prevalent in tumor tissues and can promote tumor invasion and metastasis. The XBP1-IGFBP3 signaling pathway promotes invasion and metastasis in NSCLC.^[41]^ Urra et al found that ATF4-mediated activation of lysosomal-associated membrane protein 3 promotes metastasis in breast cancer cells in a hypoxic environment, and ATF4 is upregulated in esophageal cancer, which increases matrix metalloproteinases to promote cell invasion and metastasis.^[42]^ Additionally, triple-negative breast cancer cells with high expression of XBP1 are more likely to metastasize to the lung. These findings indicate that the degree of ERS is positively correlated with the depth of infiltration and metastasis. Liu et al observed that downregulation of the ERS indicator protein HSPA5 significantly reduced the invasive capacity of tumor cells by inhibiting phosphatidylinositol 3-kinase/AKT signaling.^[43]^ Epithelial mesenchymal transition (EMT) has an important role in tumor development, and Zeindl-Eberhart et al found that EMT causes ERS. Therefore, targeting ERS could be an important strategy to control tumor progression.^[44]^

### 4.1. Head and neck cancer

Jiang et al found that sinomenine hydrochloride reversed EMT through ERS-mediated autophagy, thereby impairing the invasion of U87 and SF767 human glioblastoma cells.^[45]^

### 4.2. Digestive tract tumor

The corilagin inhibits the progression of migration and infiltration of the esophageal cancer cell lines CA109 and KYSE150 through the activation of mitochondrial and ERS signaling pathways.^[46]^ Schisandra chinensis A inhibits the proliferation, invasion, and metastasis of GES-1 human gastric epithelial cells and AGS gastric cancer cells partly through the PERK/eIF2α/CHOP signaling pathway.^[47]^

### 4.3. Gynecologic urogenital tumors

Betulinic acid (BA) induces GRP78 overexpression that activates the ERS sensor PERK. Subsequent phosphorylation of eIF2α inhibits β-catenin activation and ultimately impedes breast cancer cell invasion and migration by inhibiting c-Myc-mediated aerobic glycolysis in breast cancer cells. The lung colonization model validated that BA inhibited breast cancer metastasis in vivo.^[48]^ Sini san inhibits chronic psychological stress-induced lung metastasis and the stemness of breast cancer cells by attenuating GRP78 expression, as well as by reversing cortisol-induced stem cell-like populations and mammosphere formation.^[49]^ Matrine inhibits invasion of DU145 and PC-3 prostate cancer cells by suppressing EMT, as a result of proteasomal CT-like activity inhibition via activation of UPR/ER stress both in vitro and in vivo.^[50]^ Endoplasmic reticulum protein 29 (ERp29) is an ER-associated protein. High expression of ERp29 is negatively correlated with tumor progression and regulation of mesenchymal epithelial transition. One study found that ERp29 expression was upregulated in CaSki cervical cancer cells and significantly reduced in the presence of dihydrotanshinone IIA, suggesting that dihydrotanshinone IIA may have a particularly potent inhibitory effect on cancer cells with metastatic potential.^[51]^

## 5. Tumor treatment resistance

As previously described, ERS plays dual roles in cancer. Sustained drug use alters the tumor microenvironment, which activates both ERS and UPR. UPR regulates downstream pro-survival or pro-apoptotic signaling through 3 sensors. Cao et al summarized several studies that found that ERS-mediated pro-survival signaling plays an important role in drug resistance in antitumor therapy.^[52]^ There are various resistance mechanisms, including inhibition of apoptosis, activating protective autophagy, regulating adenosine triphosphate-binding cassette transporters, affecting Wnt/β-catenin signaling and noncoding ribonucleic acid, and triggering immunosuppression. Several studies have shown that ERS is also involved in radiotherapy resistance in cancer.^[53,54]^

### 5.1. NSCLC

Chalcomoracin is a Diels–Alder adduct derived from mulberry leaves that dose-dependently inhibits the proliferation of H460, A549, and PC-9 human lung cancer cells by enhancing ERS to increase sensitivity to radiotherapy.^[55]^ Fucoidan enhances cisplatin-induced cytotoxicity in human lung cancer cells by upregulating TLR4/CHOP-mediated activation of caspase-3 and poly-(adenosine diphosphate-ribose) polymerase.^[56]^

### 5.2. Colorectal cancer

A previous study found that the combination of curcumin and irinotecan synergistically promoted apoptosis in LoVo and HT-29 colorectal cancer (CRC) cells by increasing intracellular Ca^2 + ^and ROS and activating ERS. When CHOP is silenced in CRC cells, curcumin alone or in combination with irinotecan is unable to increase apoptosis to the same level.^[57]^ ABCG2/BCRP overexpression confers resistance to many chemotherapeutic drugs. One study found that in LoVo cells treated with lupeol, ABCG2 was decreased, ER stress marker p-eIF2α was increased, and caspase-3 was increased. These findings indicated that lupeol induces apoptosis in oxaliplatin-resistant LoVo colorectal cancer cells by altering the ERS signaling pathway through downregulation of ABCG2.^[58]^

### 5.3. Gynecologic urogenital tumors

Cai et al found that the combination of BA and PTX significantly elevated ERS by activating GRP78. GRP78 is upregulated and activates the PERK downstream signal eIF2α, which subsequently activates the transcription factor CHOP to induce apoptosis in breast cancer cells, with better synergistic effects than either alone.^[59]^ Compared with single-agent treatment, quercetin combined with PTX injection effectively inhibits PC-3 prostate cancer cell migration by downregulating hnRNPA1, making cells susceptible to PTX-induced apoptosis.^[60]^ The early and late apoptosis rates are also significantly higher in cells treated with combination therapy than in cells treated with single-agent therapy, which may be related to the increased expression of GRP78 and genes associated with ERS and ROS production and activation of the PERK-eIF2α-ATF4 pathway in combination-treated cells.

## 6. Summary

ERS is involved in the occurrence and development of many tumors, including pancreatic, esophageal, lung, liver, and ovarian cancers. As a self-protective and pro-survival regulator of tumor cells, ERS is involved in a variety of malignant biological behaviors, such as unlimited proliferation, autophagy, radiotherapy resistance, glycolytic energy regulation, immune escape, metastasis, and invasion. Therefore, characterizing the changes in ERS-related pathways during tumor pathogenesis is beneficial for diagnosis and treatment.

The antitumor pathways activated by TCM include blocking cell proliferation, promotion of apoptosis, synergistic potentiation, reversal of resistance to chemotherapeutic or targeted drugs, reduction of side effects of Western medicine treatments, and improvement of immunity. This review summarizes the positive effects of herbal compounds, single herbal medicines, major components of herbal medicines, and novel formulations via the endoplasmic reticulum pathway on tumor development and treatment of gastric cancer, colorectal cancer, liver cancer, gallbladder cancer, oral cancer, lung cancer, breast cancer, leukemia, esophageal cancer, cervical cancer, prostate cancer, malignant mesothelioma, and glioma (Table 1). Many studies have examined the effects of herbal or chemical drugs on tumorigenesis and development with regard to ERS, providing a new entry point for tumor prevention and treatment. TCM can also influence tumor progression and efficacy through the bidirectional regulation of ERS. However, TCM still occupies an auxiliary position in the clinic. This is a problem that needs to be solved, and identifying highly efficacious antitumor Chinese medicines, Chinese medicine components, or Chinese medicine preparations that can be widely used in the clinic may address this issue. The mechanism of bidirectional regulation of ERS by Chinese medicine and the balance point of bidirectional regulation need to be exhaustively explored.

**Table 1:** The recent studies on the effect of TCM on cancers via ERS.

Function	Cancer	Number	TCM	Mechanism	References
Tumor cell proliferation	Head and neck cancer	1	Glaucocalyxin A	ATF4/ CHOP/CHAC1 pathways	^[9]^
		2	Tetrandrine	GRP78 and GADD153	^[10]^
	Digestive tract tumor	3	Tanshinone IIA	BIP and CHOP	^[11]^
		4	Wogonoside	IRE1α-TRAF2-ASK1 pathways	^[12]^
		5	*Cyperus amuricus*	GRP78/BiP; CHOP-URP-PERK/IRE1/ATF6	^[13]^
		6	Muscone	PERK/ATF4/DDIT3 pathways	^[14]^
		7	Celastrol	IRE1α-XBP1s pathways; GRP78/BiP, ATF4 and CHOP	^[15]^
		8	Salidroside	p-PERK, p-eIF2a, ATF-6 and CHOP	^[16]^
		9	TTF1-NPs	GRP78, PERK, IRE1α, ATF6 and CHOP; PERK-eIF2α-ATF4 and ATF6	^[17,18]^
		10	Epigallocatechin-3-gallate	p-PERK and p-eIF2α	^[19]^
		11	Shikonin	PERK/elF2α/ATF4/CHOP, and IRE1α/JNK	^[20]^
		12	Gambogenic acid	IRE1α, BiP, p-eIF2α, and ATF4	^[21]^
		13	A purified resin glycoside fraction from pharbitidis semen	ERS-JNK pathways	^[22]^
	Gynecologic urogenital tumors	14	Saikosaponin-A	GPR78 and CHOP	^[23]^
		15	Identification of dihydrotanshinone I	PERK	^[24]^
	Non-small cell lung cancer	16	Kushenol Z	CHOP	^[25]^
		17	Evodiamine	IRE1α-TRAF2-ASK1-JNK	^[26]^
		18	flavonoid components in Astragali Radix	IRE1α-XBP1s	^[27]^
	Others	19	Shikonin	p-eIF2α and CHOP	^[28]^
		20	Psoralen	CHOP, IREa, XBP-1s, GRP78, PERK and ATF-6	^[29]^
		21	Artesunate	ATF4-CHOP-CHAC1	^[30]^
		22	(-)- Epigallocatechin-3-gallate	GRP78, ATF4, XBP1s, CHOP and EDEM	^[31]^
		23	Asperuloside	GRP78, p-PERK, p-eIF2α, CHOP, p-IRE1, XBP1, and ATF6	^[32]^
Tumor cell autophagy	Non-small cell lung cancer	24	Ginsenoside	ATF4-CHOP-AKT1-mTOR	^[36]^
		25	Guangsangon E (GSE)	ROS-ERS (GRP78, IRE1α and ATF4)	^[37]^
	Colorectal cancer	26	Serpentine	GRP78, p -PERK, p-elF2α and CHOP	^[38]^
		27	Cryptotanshinone	Cell autophagy by ERS	^[39]^
	Gynecologic urogenital tumors	28	Cucurbitacin-I	IRE1α-caspase 1-p-PERK-eIF2-ATF4-CHOP	^[40]^
Tumor cell invasion and metastasis	Head and neck cancer	29	Sinomenine hydrochloride	Reverse EMT through autophagy via ERS	^[45]^
	Digestive tract tumor	30	Corilagin	GRP78-caspase-7 and caspase-12	^[46]^
		31	Schizandrin A	PERK/eIF2α/CHOP pathways	^[47]^
	Gynecologic urogenital tumors	32	Betulinic acid	GRP78-PERK-p-eIF2α-β-catenin and c-Myc	^[48]^
		33	Sini San	GRP78-chronic psychological stress-Wnt/β-catenin	^[49]^
		34	Matrine	PERK-p-eIF2α-ATF4-CHOP pathways-E-cadherin	^[50]^
		35	Tanshinone IIA	PERK-eIF2α-ATF4-CHOP/IRE1-ASK1-P38	^[51]^
Tumor treatment resistance	Non-small cell lung cancer	36	Chalcomoracin (CMR)	BiP and CHOP	^[55]^
		37	Fucoidan	TLR4/CHOP-cleaved caspase-3-PARP	^[56]^
	Colorectal cancer	38	Curcumin	PDI and CHOP	^[57]^
		39	Lupeol	p-eIF2α	^[58]^
	Gynecologic urogenital tumors	40	Betulinic acid	GRP78-PERK and eIF2α-CHOP	^[59]^
		41	Quercetin	CHOP and GRP78	^[60]^

Akt = Akt serine/threonine kinase, ASK1 = apoptosis signal-regulating kinase 1, ATF = activating transcription factor, BiP = lumenal-binding protein, CHAC1 = ChaC glutathione specific gamma-glutamylcyclotransferase 1, DDIT3 = deoxyribonucleic acid damage inducible transcript 3, eIF2α = eukaryotic translation initiation factor 2 subunit alpha, EMT = epithelial mesenchymal transition, ERS = endoplasmic reticulum stress, GRP78 = glucose regulated protein 78, IRE1α = inositol-requiring enzyme-1α, JNK = c-Jun NH2-terminal kinase, mTOR = mechanistic target of rapamycin kinase, PARP = poly-(adenosine diphosphate-ribose) polymerase, PERK = protein kinase R (PKR)-like endoplasmic reticulum kinase, ROS = reactive oxygen species, TCM = traditional Chinese medicine, TRAF = TNF receptor-associated factor, TTF1-NPs = 5,2’,4’-trihydroxy-6,7,5’-trimethoxyflavone nanoparticles, XBP1 = X-box binding protein 1.

## Author contributions

FX, SS, LX, XX, and GH analyzed the data. FX, SS, and CX wrote the paper. CX and HW reviewed the paper.

**Conceptualization:** Fan Xia.

**Data curation:** Fan Xia, Suling Sun.

**Formal analysis:** Hongzhi Wang.

**Investigation:** Fan Xia, Suling Sun, Li Xia.

**Methodology:** Xiuli Xu, Ge Hu.

**Project administration:** Hongzhi Wang, Xueran Chen.

**Resources:** Xiuli Xu.

**Software:** Ge Hu.

**Supervision:** Li Xia, Hongzhi Wang.

**Writing – original draft:** Fan Xia.

**Writing – review & editing:** Xueran Chen.
